# A Comparative Study for License Application Regulations on Proprietary Chinese Medicines in Hong Kong and Canada

**DOI:** 10.3389/fmed.2021.617625

**Published:** 2021-03-09

**Authors:** Linda L. D. Zhong, Wai Ching Lam, Fang Lu, Xu Dong Tang, Aiping Lyu, Zhaoxiang Bian, Heather Boon

**Affiliations:** ^1^Hong Kong Chinese Medicine Clinical Study Centre, Hong Kong Baptist University, Hong Kong, China; ^2^School of Chinese Medicine, Hong Kong Baptist University, Hong Kong, China; ^3^Leslie Dan Faculty of Pharmacy, University of Toronto, Toronto, ON, Canada; ^4^Department of Epidemiology, Harvard T.H. Chan School of Public Health, Boston, MA, United States; ^5^China Academy of Chinese Medical Sciences, Beijing, China

**Keywords:** proprietary Chinese medicine, traditional Chinese medicine, herbal medicine, natural health products, Chinese medicine regulation, Chinese medicine registration, product license application regulation, Zhizhu Kuanzhong

## Abstract

**Ethnopharmacological Relevance:** Chinese Medicine plays a symbolic role among traditional medicines. As Chinese Medicine products are widely used around the globe, regulations for Chinese Medicine products are often used as models for the efficient regulation of natural products that are safe, and high-quality.

**Aim of the Study:** We aimed to compare the regulatory registration requirements for Proprietary Chinese Medicines in Hong Kong and Canada.

**Materials and Methods:** We compared registration requirements for Proprietary Chinese Medicine in Hong Kong and Canada based on publicly available information provided by the respective Regulators. A marketed product, Zhizhu Kuanzhong Capsule (SFDA approval number Z20020003; NPN approval number 80104354), was used as a case study to demonstrate the similarities and differences of the requirements in both Hong Kong and Canada.

**Results:** There were similarities and differences between the two regulatory systems in terms of the quality, safety and efficacy requirements. Despite the superficial appearance of similar categories and groups/classes, Hong Kong requires significantly more primary test data compared to Canada's reliance on attestation to manufacturing according the standards outlined in approved reference pharmacopeias/texts.

**Conclusion:** Improved understand of the similarity and differences will enable applicants to plan appropriate strategies for gaining product approval. Exploring ways to harmonize the regulatory process has the potential to benefit manufacturers, regulators, and patients by increasing efficiency and decreasing costs.

## Introduction

Regulation of health products is generally considered necessary to protect the public. The vast majority of countries have implemented policies and practices aimed at reviewing the safety and effectiveness of health products before allowing them to be marketed to the general population. In an increasingly globalized marketplace, manufacturers face a wide array of regulatory regimes as each country requests different information or evidence and often requires different standards for assessing products. While recognizing the right of each sovereign nation to set its own standards, one wonders whether voluntary harmonization of key standards and evidence requirements might save significant time and money ultimately increasing access of populations to safe, effective and high quality products.

The implementation of evidence-based reviews to avoid costly replication of efforts and facilitate timely access to medications has been a topic of global discussion and debate. For example, in the United States, the *Prescription Drug User Fee Act VI* and *21st Century Cures Act* recommend a more effective and efficient regulatory review model, authorizing the Food and Drug Administration to enhance capacity to review products under a variety of models and pathways (1). In the European Union, the European Medicines Agency has been taking steps toward more flexible approaches to its drug approval system (2).

Traditional medicine is defined by the World Health Organization as “the sum total of the knowledge, skill, and practices based on the theories, beliefs, and experiences indigenous to different cultures, whether explicable or not, used in the maintenance of health as well as in the prevention, diagnosis, improvement or treatment of physical and mental illness” (3). Traditional medicine products often have long histories of use in their original cultural settings but may have limited translational research supporting their use. As demand for these products increases around the world, especially in markets outside the country of origin, there is an urgent need for regulation of traditional medicines. This creates challenges for regulators and manufactures. Regulators look for conventional scientific evidence of safety and efficacy, while consumers and manufacturers are concerned that over regulation may limit patient access to safe and effective medicines that have been used for hundreds of years in their country of origin. Unlike Western biomedicine, health claims of traditional medicines are sometimes difficult to evaluate in jurisdictions outside the country of origin where there may be limited experience or expertise in assessing the quality of traditional products. In addition, the significant variation in guidelines and requirements for product registration/licensing creates unnecessary barriers for manufacturers.

In order to explore these challenges, we conducted a case study of the regulatory requirements for obtaining a product license for Zhizhu Kuanzhong Capsule, a Proprietary Chinese Medicine in two policy environments: Hong Kong and Canada. Hong Kong and Canada were chosen because they both have well-developed regulatory systems for traditional medicines, but are based in very different cultures which allows the identification of similarities and differences that may have relevance for a range of systems. We focused on the quality control, safety, and effectiveness aspects of the regulatory process as these are arguably more amenable to possible harmonization across states and cultures. The paper concludes with a discussion of components of the regulatory process where harmonization may have the greatest impact to facilitate the efficiency of the regulatory approval process while also enhancing its rigor and ability to achieve the objective of protecting the public.

## Definition and Regulations

The regulatory pathway and requirements for Proprietary Chinese Medicine in Hong Kong and Canada are available online on their respective regulatory bodies' websites (4–7).

**Figure 1:**
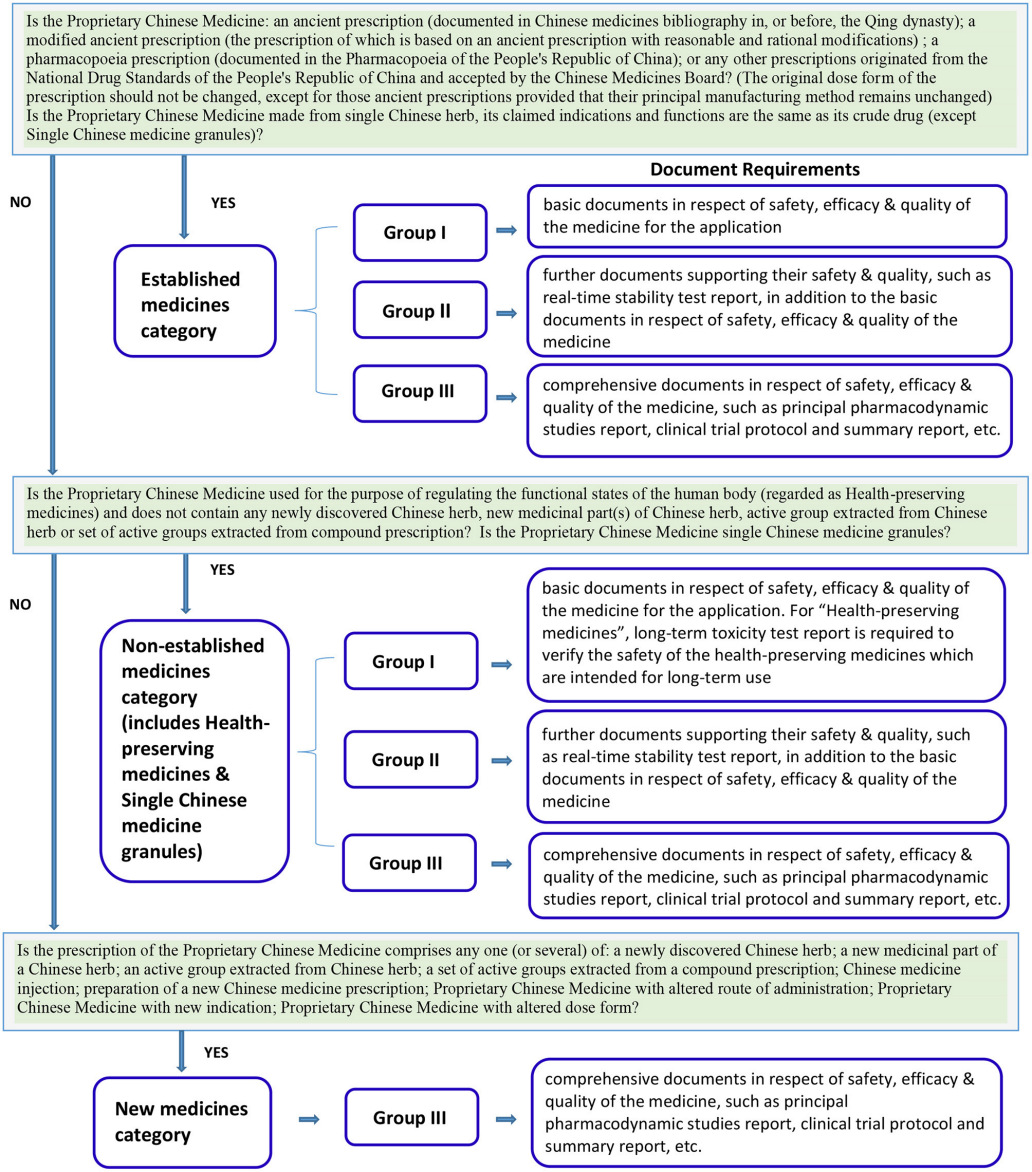
The classification categories and registration groups of Proprietary Chinese Medicines and their requirements in Hong Kong (8).

In Hong Kong, Proprietary Chinese Medicine is regulated by Chinese Medicine Council of Hong Kong, a statutory body established under the Chinese Medicine Ordinance established since 1999. Under the regulation, the selection of the classification category and the registration group of a Proprietary Chinese Medicine product license application is a decision made by the applicant and reviewed by the regulatory body. The registration requirements are dependent on the selected classification category and registration group (Figure 1). Other than the “New Medicine Category” that must be registered under Group III, for Proprietary Chinese Medicines under the “Established medicines category” (ancient or pharmacopeia prescriptions with original dose form) and “Non-established medicines category” (health-preserving medicines and single Chinese medicine granules), there are no distinctive guidelines which help the applicant to determine whether products should be registered under Group I, Group II or Group III. The applicants may choose to apply for registration in any of the three groups (8). This creates an incentive for applicants to select registration groups with fewer requirements, i.e., Group I with basic documents or Group II with further safety and quality supporting documents, instead of Group III which requires the submission of a dossier with comprehensive documents (see Table 1 for a summary of required materials). Though the applicants are encouraged to consult the Chinese Medicine Regulatory Office of the Department of Health, currently there is no official pre-submission meeting arrangement in Hong Kong to help ensure that the applicants have selected the right product license registration group.

**Table 1:** Product license application requirements summary for Hong Kong and Canada (5, 9).

		**Group I/Class I**	**Group II/Class II**	**Group III/Class III**
Hong Kong	Established medicines category	Basic documents **Product safety documents** 1. Heavy metals and toxic elements test report 2. Pesticide residues test report 3. Microbial limit test report 4. Acute toxicity test report 5. Long-term toxicity test report 6. Local toxicity test report 7. Summary report on product safety documents **Product efficacy documents** 1. Interpretation and principle of formulating a prescription 2. Reference materials on product efficacy 3. Summary report on product efficacy documents **Product quality documents** 1. Manufacturing method 2. Physicochemical properties of crude drugs 3. Product specification, method and certificate of analysis 4. Accelerated stability test report or general stability test report	Further documents **Product safety documents** 1. Heavy metals and toxic elements test report 2. Pesticide residues test report 3. Microbial limit test report 4. Acute toxicity test report 5. Long-term toxicity test report 6. Local toxicity test report 7. Mutagenicity test report 8. Carcinogenicity test report 9. Reproductive and development toxicity test report 10. Summary report on product safety documents **Product efficacy documents** 1. Interpretation and principle of formulating a prescription 2. Reference materials on product efficacy 3. Summary report on product efficacy documents **Product quality documents** 1. Manufacturing method 2. Physicochemical properties of crude drugs 3. Product specification, method and certificate of analysis 4. Real-time stability test report	Comprehensive documents **Product safety documents** 1. Heavy metals and toxic elements test report 2. Pesticide residues test report 3. Microbial limit test report 4. Acute toxicity test report 5. Long-term toxicity test report 6. Local toxicity test report 7. Mutagenicity test report 8. Carcinogenicity test report 9. Reproductive and development toxicity test report 10. Summary report on product safety documents **Product efficacy documents** 1. Interpretation and principle of formulating a prescription 2. Reference materials on product efficacy 3. Principal pharmacodynamic studies report 4. General pharmacological studies report 5. Clinical trial protocol and summary report 6. Summary report on product efficacy documents **Product quality documents** 1. Manufacturing method 2. Physicochemical properties of crude drugs 3. Product specification, method and certificate of analysis 4. Real-time stability test report
	Non-established medicines category			
	New medicines category			
Canada	Compendial (NHPD) Monograph)	- NNHPD Label text - Evidence (Attestation to a NNHPD monograph) - Animal Tissue form - Finished Product Specifications (Upon request only)		
	Traditional		- NNHPD Label text - Quality Summary Report: Characterization, identification and quantification standards; Purity standards General indicators for quality and Performance Tests - Safety Evidence: extensive history of use; Cautions, warnings and contra-indications; no new, unknown safety concerns have been identified. Efficacy Evidence: Pharmacopoeial Evidence- a NNHPD monograph or from a recognized pharmacopeia; Minimum of two other traditional references - Independent references; authoritative references and from a reputable source; an expert opinion as second reference. - Animal Tissue form - Finished Product Specifications	Full assessment required: - NNHPD Label text - Quality Summary Report: Characterization, identification and quantification standards; Purity standards General indicators for quality and Performance Tests - Safety Evidence: extensive history of use; Cautions, warnings and contra-indications; no new, unknown safety concerns have been identified. Efficacy Evidence: Pharmacopoeial Evidence - a NNHPD monograph or from a recognized pharmacopeia; Minimum of two other traditional references - Independent references; authoritative references and from a reputable source; an expert opinion as second reference. - Animal Tissue form - Finished Product Specifications
	General (Non-traditional)		Low or Medium risk Proprietary Chinese Medicine - NNHPD Label text - Quality Summary Report: Characterization, identification and quantification standards; Purity standards General indicators for quality and Performance Tests - Evidence/Safety Summary Report: Phase II clinical trials; Epidemiological studies; Pilot and open label studies; Reputable textbooks; Systematic review other than meta-analysis; Published, peer-reviewed, detailed narrative reviews which cite detailed primary evidence; Published compilations referring to traditional use etc., - Evidence (Minimum two pieces) - Animal Tissue form - Finished Product Specifications	High risk Proprietary Chinese Medicine, full assessment required: - NNHPD Label text - Quality Summary Report: Characterization, identification and quantification standards; Purity standards General indicators for quality and Performance Tests - Evidence/Safety Summary Report: NNHPD published monographs; Phase III or phase IV clinical trials (randomized, controlled, well-designed); Meta-analysis (controlled and well-designed); Prospective observational studies or combinations of one prospective study and one retrospective study; Evidence of a positive decision from another regulatory agency - Evidence (Minimum two pieces) - Animal Tissue form - Finished Product Specifications

**Figure 2:**
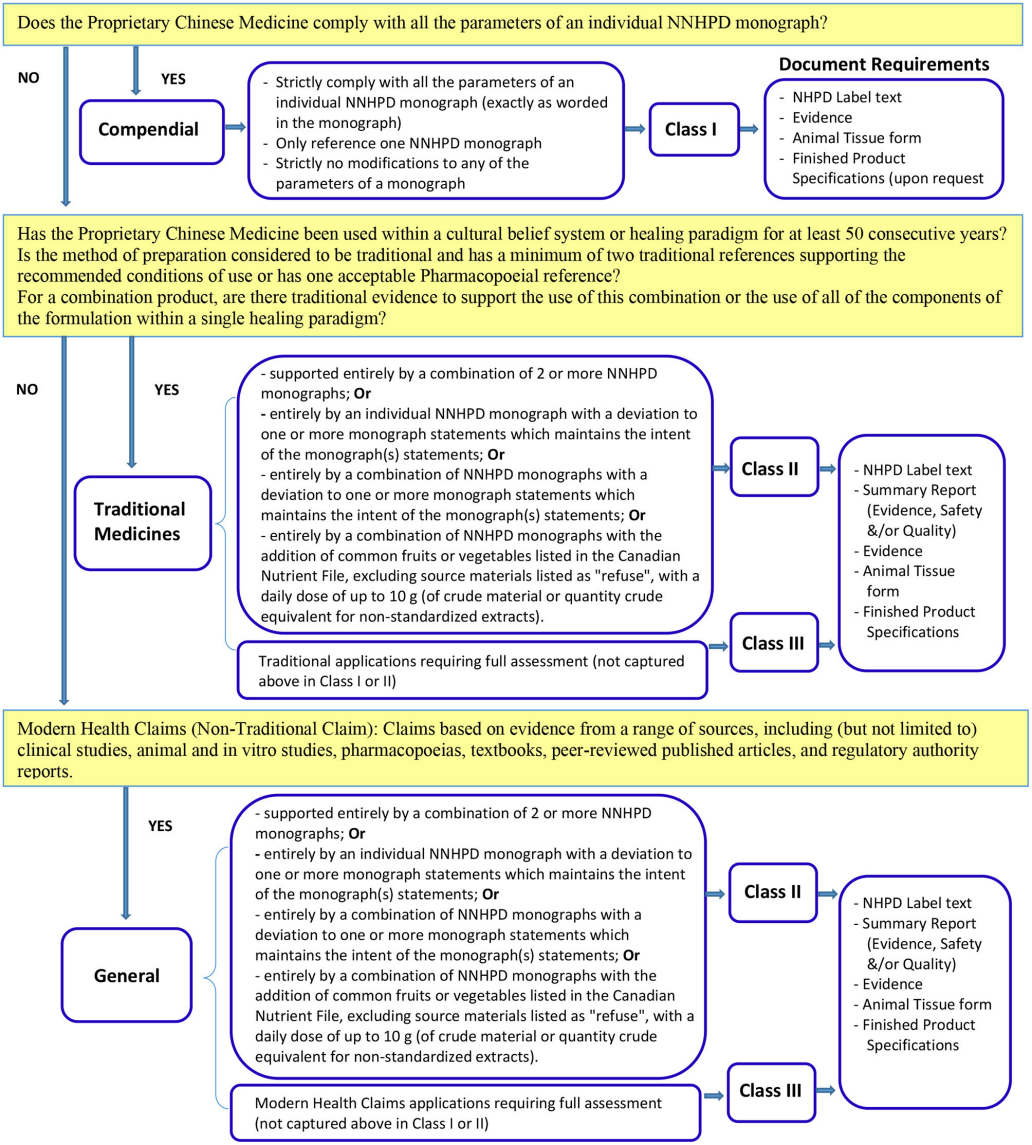
The types and classes of Proprietary Chinese Medicines product license application and their requirements in Canada (9).

In Canada, Proprietary Chinese Medicine is defined as a natural health product (NHP) and regulated by Natural and Non-prescription Health Products Directorate of Health Canada under the Natural Health Products Regulations which came into effect on January 1, 2004. A wide range of health products whose active ingredients exist in nature and are used for self-limiting conditions fall within the category of natural health products including herbal medicines, vitamins and minerals, essential fatty acids and many different traditional medicines including most Proprietary Chinese Medicine. Proprietary Chinese Medicine can be registered under a Class I or Class II or Class III product license (9) (see Figure 2). For example, Proprietary Chinese Medicines which meet the Compendial requirements may submit an application under Class I. The well-defined application guidelines help applicants to determine the right type and class of the product license for which they should apply. If in doubt, applicants can request a Pre-submission Meeting to clarify the type of application required (9). In addition, if after reviewing all relevant regulations, guidance and tools, an applicant is unsure if a product is suitable for licensing as a natural health product, the applicant is encouraged to submit a product classification request prior to submitting a product licensing application. It should be noted that in the Canadian system, at least one “claim” must be approved as part of the licensing application. Recognizing that many Proprietary Chinese Medicines may be used for a wide variety of conditions (representing different claims), the regulatory system, which requires additional documentation for applications in Classes 2 and 3, creates an incentive for applicants to seek approval for a single claim in the lowest Class possible with the least regulatory burden.

## Structured (Quality, Safety, and Efficacy) Requirements of Proprietary Chinese Medicines in Hong Kong and Canada

### Hong Kong

For Proprietary Chinese Medicines in the “Established medicines category” and “Non-established medicines category” to be registered under Group I, basic documents related to safety, efficacy & quality of the medicine are required for the application. For “Health-preserving medicines” in the “Non-established medicines category,” a long-term toxicity test is required to verify safety because these products are intended for long-term use.

For Proprietary Chinese Medicines in the “Established medicines category” and “Non-established medicines category” to be registered in Group II, further documents supporting their safety and quality are required, such as real-time stability testing, in addition to the basic documents related to the safety, efficacy, and quality of the medicine.

For Proprietary Chinese Medicines under the “Established medicines category,” “Non-established medicines category,” and “New medicines category” to be registered in Group III, comprehensive documents documenting the safety, efficacy, and quality of the medicine are required, such as principal pharmacodynamic studies, clinical trial protocol, and summary report, etc. (8).

### Canada

The Natural and Non-prescription Health Products Directorate (NNHPD) requests that applicants identify the class under which they are applying in the cover letter of their application according to the definitions. If a Class is not identified correctly in the cover letter, the application will be refused.

By attesting to a monograph, the applicant is confirming that the application meets all of the monograph parameters (Class I) to which the applicant has attested. If applicants are not attesting to full monograph parameters in Class II or III applications, they must ensure that evidence or a rationale for not attesting to the monograph has been provided.

The details of the quality, safety and efficacy requirements for Hong Kong and Canada are listed in Tables 2–4, respectively (10–16).

**Table 2:** The quality requirements of proprietary Chinese medicines in Hong Kong and Canada (10, 11).

**Requirements**	
**Hong Kong**	**Canada**
**INDIVIDUAL PRODUCT QUALITY DOCUMENT**	
**1. Manufacturing Method** (1) Processing procedure for each raw herb (2) Names and quantities of all excipients used in the processes (3) Specified technical controls for procedures that may affect the quality of the Proprietary Chinese Medicine	**1. Characterization** **A. Chemicals** **B. Processed ingredients** (1) Process characterization of crude materials (2) Process characterization for highly processed ingredients **C. Extracts** (1) Standardized extracts (2) Fortified extracts
**2. Physicochemical Properties of Crude Drugs** **A. The crude drug(s) of a Proprietary Chinese Medicine** (1) A newly-discovered Chinese herb (2) A new medicinal part of a Chinese herb (3) An active group extracted from Chinese herb (4) A set of active groups extracted from a compound prescription (i) Description (ii) Identification Method (iii) Inspection (iv) Assay **B. Crude herbs of the Proprietary Chinese Medicine that do not fall into any of the four groups as mentioned above in (A)**	**2. Physicochemical Properties of Raw Material** **A. Identification Tests of the ingredients** (1) Appropriate Identification of botanical products (2) Appropriate identification of specific medicinal ingredients (3) Identity testing on the finished product **B. Quantity** (1) Quantification by assay (2) Quantification by input **C. Purity Standards** **D. Additional Tests & Criteria** (1) General Indicators for Quality (2) Performance Tests (3) Analytical testing and requirements to support label claims (4) Reduced testing schedules that are captured on specifications (5) Antimicrobial effectiveness testing Where an NNHPD monograph exists for an ingredient, the specification section of the monograph should be consulted
**3. Finished Product Specification, Methods & Certificate of Analysis** **A. Product specification** (1) Description (2) Identification (3) Assay (4) Inspection **B. Test Methods** **C. Test Report**	**3. Finished Product Specification and Test Methods** **A. Product specification** (1) Physical description (2) Identity testing on the finished product (3) The finished product specifications **B. Test Methods**
**4. Stability Test Report**	**4. Stability Test Report**
**REQUIREMENTS FOR TEST LABORATORIES**	
(1) Met the requirements set by the International Standardization Organization (i.e. ISO/IEC 17025) (2) Good Laboratory Practice (GLP) (3) Other laboratories accepted by the Board	Good Laboratory Practice (GLP)

*Detailed requirements are listed in Supplementary Material 1*.

**Table 3:** The safety requirements and purity standards (under Quality Requirements' guidelines) of proprietary Chinese medicines in Hong Kong and Canada respectively (11, 12).

**Hong Kong (Safety requirements)**	**Canada (Purity standards)**
Documents supporting the safety claims of the product are required to be submitted to the Chinese Medicines Board for assessments. The documents shall include the basic and toxicological tests. Other reference material may be provided to support the safety claims of the products, e.g., the published bibliography or monographs, etc.	Safety Requirements: - supported by its history of use (at least 50 consecutive years of traditional use within a cultural health system or paradigm) and no new, unknown safety concerns outside of evidence for traditional use. Two independent references - Modern Health Claims are based on the identified risks to health. Evidence recommendations are categorized into low, medium, and high risk. Within any risk category, the evidence may be sufficient to support both safety and efficacy when it is appropriate for the claim and when it fully reflects the product's recommended conditions of use. For the low and medium categories, methodologically weak safety evidence should be supplemented to demonstrate consistency in results and plausibility. For high risk category, product specific evidence is recommended with a complete critical summary reflecting the totality of evidence that usually reflect more than one type of evidence. Due to the test requirements' similarity, in this comparative study we compare the safety requirements of Proprietary Chinese Medicine product in Hong Kong to the product's purity standard in Canada.
**A. Heavy metals and toxic element test** Heavy metals (mercury, lead & cadmium) or toxic elements (arsenic)	**A. Chemical Contaminants** Elemental impurities (Catalysts and environmental contaminants); Topical products
**B. Pesticide residues test**	**B. Pesticide residues**
**C. Microbial limit test** Total aerobic count, molds & yeast count & the presence of specified bacteria	**C. Microbial Contaminants** Multi-Component products, products in liquid dosage form.
**D. Acute toxicity test** Median lethal/Maximum tolerable dose	**D. Other Impurities** (1) Mycotoxins (e.g., aflatoxins) Testing (2) Cyanobacterial toxins (e.g., microcystins) (3) Solvent residues (4) Hormone testing of animal materials (5) Incidental impurities, related substances and process impurities (6) Potential adulterants in natural health products
**E. Long-term toxicity test**	
**F. Local toxicity test** Local dermal toxicity/Mucous membrane irritation test	
**G. Mutagenicity test** For Group II & Group III application to examine the carcinogenicity or reproductive toxicity: Bacterial reverse mutation test, chromosomal aberration test with mammalian cells in culture & micronucleus test with rodents	
**H. Carcinogenicity test** For Group II & Group III application to examine the carcinogenicity or tumorigenicity: Preliminary carcinogenicity study & full-scale carcinogenicity study	
**I. Reproductive and development toxicity test** For Group II & Group III application only, to examine any toxic effects on animal's reproductivity and teratogenic effect on their offspring: General reproductive toxicity, teratogenicity & perinatal toxicity test	
**J. Requirements for test laboratories** International Standardization Organizations, Chinese Medicines Board, State Food & Drug, or Chinese Medicines Board	**E. Requirements for test laboratories** - Good Laboratory Practice (GLP)
**K. Summary report of product safety documents** Overall conclusion; a reasonable assessment	**F. Summary report of product safety documents** Safety Overview; risk Information and Risk Mitigation

*Detailed requirements are listed in Supplementary Material 2*.

**Table 4:** The efficacy requirements of proprietary Chinese medicines in Hong Kong and Canada (11, 13).

**Requirements**	
**Hong Kong**	**Canada**
**1. Reference materials on product efficacy** Including reference literature or documentary proofs on long history of use (i) Established medicines category (ii) Non-established medicines category - Health-preserving medicines - Single Chinese medicine granules (iii) New medicines category	**1. Evidence requirements for safety and efficacy** The safety and efficacy of health claims associated with NHPs must be supported by appropriate evidence such as clinical trial data to references to published studies, journals, pharmacopeias and traditional resources. The type and amount of supporting evidence required is dependent on the proposed health claim of the product and its overall risks. The evidence requirements for efficacy are listed depending on whether the product is a: - Traditional Medicine; - NHP with Traditional use claims or with Modern Health Claims
**2. Interpretation and principle of formulating a prescription** - written by professionals - efficacy and safety - General requirements: Source, ingredients, specified usage and dosage of the preparation, functions & indications, interpretation, precautions (if any)	**2. Efficacy Evidence for Traditional Medicines** **A. Pharmacopoeial Evidence for Traditional Medicines** - relevant pages of a monograph from a recognized pharmacopeia; - a monograph published by a reputable agency with a definition of traditional medicines comparable to that of the NNHPD **B. Other Types of Efficacy Evidence for Traditional Medicines** at least 2 independent references that support the recommended conditions of use; an expert opinion if only 1 reference
**3. Principal pharmacodynamic studies** For Group III application only: Brief requirements, requirements for test laboratories	**3. Efficacy Evidence Recommendations for NHPs with Modern Health Claims** **A. Efficacy Evidence for the High-Risk Category** complete critical summary; systematic review; demonstrate statistically significant outcomes; additional evidence **B. Efficacy Evidence for the Medium Risk Category** Evidence as individual references: **C. Efficacy Evidence Requirements for the Low Risk Category** For minor health conditions & diseases; treatment of symptoms or risk factors of serious or major conditions or the risk reduction of these conditions; general health maintenance, support, or promotion that refers to modification of a biochemical or physiological function of a nutritional nature or implies benefit to a minor disease or health condition. Evidence to reflect the low-risk nature This category includes most vitamins, minerals, essential nutrients, and other nutrients recommended for use by healthy adults.
**4. General pharmacological studies** For Group III application only: Brief requirements, requirements for test laboratories	
**5. Clinical trial protocol and summary report** For Group III application only. (i) Brief requirements (a) Phases of clinical trials (b) Contents of clinical trial protocol (c) Contents of the summary report of clinical trials (d) Documents to be submitted upon application (ii) Requirements for Clinical Trial centers	
**6. Summary report on product efficacy documents** An overall conclusion and a reasonable assessment of product efficacy.	**4. Summary report on product efficacy documents** Consists of: recommended use or purpose (health claim); critical overview organized based on the claims of the product; dosage and Other Conditions of Use

*Detailed requirements are listed in Supplementary Material 3*.

## Impact of Regulations in Hong Kong and Canada

Product regulations are the gates defending public health against health products without proven quality, safety, and effectiveness. They should ensure minimum quality of products provide the basis for quick action if post marketing reports identify issues with specific products. Although there is no international standard for regulation for Proprietary Chinese Medicines, the regulatory authorities of Hong Kong and Canada have both adopted a multi-pronged regulatory strategy by dividing applications into groups and classes.

In Hong Kong since registration of Proprietary Chinese Medicines took effect in 2003, more than 2,400 Proprietary Chinese Medicines products have been issued a certificate of registration (17). The registered products not only provide safe Chinese Medicine products to the public, but also have driven ancillary economic activities and created employment opportunities. In 2019, the Hong Kong government established the Hong Kong Chinese Medicine Development Fund with HKD 500 million to promote the development of the Chinese Medicine including support to enhance the overall standard of the industry (18). Manufacturers of Proprietary Chinese Medicines can apply the fund to design or purchase equipment that meets the requirements of Chinese Medicine Manufacturing Quality Management (GMP) (19). Moreover, the government will provide a list of accredited testing institutes to assist manufacturers to meet technical requirements and provide required documentation throughout the registration process (20).

In 2012, Canada established guidelines regarding product license application for traditional medicines including Chinese Medicine. Noteworthily, instead of evidence for ingredients that are already known to be safe and efficacious, Canada's regulatory approach allows assessment of traditional medicines based on efficacy and safety data from relevant traditional healing paradigms (21). However, Proprietary Chinese Medicines that consist of modified or inconsistent Chinese Medicine classical formulae are not eligible for this regulatory pathway and require detailed safety or efficacy documentation. For products that are eligible for Class I, manufacturers must attest that they are manufacturing the product according to the requirements outlined in the Canadian monograph which is based on the Canadian regulatory authority's review of the evidence and determination of what is required to ensure a product is safe and of sufficient quality (22).

## Taking One Proprietary Chinese Medicine as an Example: Zhizhu Kuanzhong (ZZKZ) Capsule

ZZKZ Capsule (SFDA approval number Z20020003; NPN approval number 80104354), manufactured by Shuangren Pharmaceutical Co., Ltd. of Lonch Group, has been marketed for more than 10 years in China. This Proprietary Chinese Medicine originates from the traditional prescription “Zhizhu Decoction.” ZZKZ Capsule is mainly composed of the following four commonly used Chinese herbs: Rhizoma Atractylodis Macrocephalae (plant *Atractylodes macrocephala* Koidz.), Fructus Aurantii Immaturus (plant *Citrus* × *aurantium* L.), Radix Bupleuri (plant *Bupleurum chinense* DC.), and Fructus Crataegi (plant *Crataegus pinnatifida* Bunge). The combination of these four herbs is commonly used to treat spleen deficiency, qi stagnation, liver-stomach disharmony as well as stomach duct and abdomen fullness within the traditional Chinese medicine paradigm (23). To illustrate the similarities and differences between the two regulatory processes, we summarized the requirement of documents for ZZKZ Capsule to register in both Hong Kong (Group I) and Canada (Class I) in Table 5.

**Table 5:** Zhizhu Kuanzhong Capsule - product license application in Hong Kong and Canada (8, 9).

**No**.	**Requirements**	**Hong Kong (Application no.: Z20020003)**	**Canada (Application no.: 80104354)**
1	Product License Application Category	Established medicines category	Compendial - Traditional
2	Product License Application Group	Group I	Class I
3	Product License Application Form	✓	✓
4	Evidence	**Product safety documents** 1. Heavy metals and toxic elements test report 2. Pesticide residues test report 3. Microbial limit test report 4. Acute toxicity test report 5. Long-term toxicity test report 6. Local toxicity test report 7. Summary report on product safety documents **Product efficacy documents** 1. Interpretation and principle of formulating a prescription 2. Reference materials on product efficacy 3. Summary report on product efficacy documents **Product quality documents** 1. Manufacturing method 2. Physicochemical properties of crude drugs 3. Product specification, method and certificate of analysis 4. Accelerated stability test report or general stability test report	Attest to a NNHPD Product Monograph from the Compendium of Monographs: Traditional Chinese Medicine Ingredients
5	Label text	✓	✓
6	Animal Tissue form	X	X

Regulation in Hong Kong requires six different safety test reports including heavy metals and toxic elements report, pesticide residues report, microbial limit test report, etc. A summary of evidence including reference materials is required to support product efficacy. In addition, details of the manufacturing method, product specifications and stability testing are required.

In contrast, regulation in Canada requires confirmation that all ingredients are listed in the relevant tables of acceptable Traditional Chinese Medicine Ingredients in the Compendium of Monographs and attestation from the manufacturer that the product is manufactured according to the preparations and methods of processing outlined in one of five specific approved reference pharmacopeias/texts. Similarly, conditions of use and adverse effects identified on the label must be consistent with those outlined in one of the five acceptable reference pharmacopeias (24).

Thus, Hong Kong and Canada take very different approaches to the regulation of the same product which appear to entail very different amounts of effort, time and money on the part of manufacturers who wish to obtain a license to market the same product. This creates a “natural experiment” that would be worth investigating to explore which regulatory approach is the most efficient at supporting the licensing of safe, effective and high quality products available for consumers.

## Discussion

One of the biggest challenges facing manufacturers is the lack of consistency of regulatory requirements globally. In some cases, the documentation required is very similar; in other cases, it varies dramatically. The classification criteria for Proprietary Chinese Medicines registration are different in Hong Kong and Canada but both involve judgements based on historic records of Proprietary Chinese Medicines and include evidence of safety, efficacy and quality. What differs is the type of evidence that must be submitted.

For example, for Proprietary Chinese Medicines that qualify for registration in Group I (Hong Kong), manufacturers are asked to provide safety and efficacy data including product specification, toxicity test report and stability test report. In contrast, for a similar category of products (Class I) in Canada, manufacturers are only required to submit documentation that the Proprietary Chinese Medicines will be manufactured and used in line with traditional data and in strict compliance with all the parameters of a specific NNHPD monograph recognized by the Health Canada. It includes the information from Pharmacopeia published by a reputable agency (e.g., the Pharmacopeia of the People's Republic of China or translated version of the Drug Standard of People' Republic of China), ensures that there are sufficient data to demonstrate the quality, safety and efficacy of the Proprietary Chinese Medicines.

Applications for Proprietary Chinese Medicines that fall into the “non-established” or “new medicines” category in Hong Kong, and under the “modern health claims” category in Canada, will be assessed under the requirements outlined for Group III in Hong Kong and under requirements of Class II or Class III in Canada. The requirements in the two countries for these kinds of products also differ. For example, in Hong Kong, clinical trials with the new indications will required to be conducted in the Hong Kong population before registration. In Canada, depending on the health risk of the Proprietary Chinese Medicine, the claimed therapeutic functions or efficacy must be supported by evidence from the health care literature (including traditional Chinese medicine literature), by research studies assessing product efficacy including clinical trials, and/or a complete critical summary of a systemic review reflecting the totality of evidence.

The safety requirements for chemical contaminants such as heavy metals and toxic elements, pesticide residues and microbial contaminants are similar in Hong Kong and Canada, but there are important differences (Supplementary Material 2). For example, Hong Kong has put emphasis on the some toxicity tests including the mutagenicity test and the reproductive and development toxicity test, as well as dose related toxicity tests; whereas Canada has more emphasis on testing for impurities that may cause toxicity, such as mycotoxins, solvent residues and incidental impurities, related substances and process impurities. Canada also has listed clear guidelines on the performance tests of the finished products (11, 12). These differences significantly increase the time and cost burden to manufactures hoping to license/register products in multiple jurisdictions.

Finally, Proprietary Chinese Medicines manufacturers raise concerns about inconsistent requirements from regulatory authorities with respect to packaging and labeling (25). In 2020, the regulatory body in Hong Kong updated guidelines regarding labels and package inserts of Proprietary Chinese Medicines (26, 27) while requirements on labeling and packaging for Proprietary Chinese Medicines are comparatively less specified in Canada (28).

As a component of traditional medicine, Proprietary Chinese Medicines create unique challenges for regulators in countries outside those where the products are traditionally used due to their complex nature and use within health paradigms that differ from Western biomedicine. It appears that safety and quality parameters may be the easiest to think about harmonizing as these parameters may be the least impacted by health paradigm differences. Agreement across regulators regarding which tests are required/acceptable to prove safety and quality would significantly increase the efficiency of the regulatory process for both manufacturers and regulators (Supplementary Material 3). With the development of global standards on submission, review and authorization of these products, manufacturers could submit the same testing data to multiple regulatory agencies. And regulatory agencies could consider fast tracking the review of products that were already approved in other countries that share similar standards.

## Conclusion

Knowledge of the Proprietary Chinese Medicines product license application procedure and requirements in Hong Kong and Canada, and understanding their similarity and differences will enable the applicants to develop an appropriate strategy for gaining product approval. Exploring ways to harmonize the regulatory process has potential to benefit manufacturers, regulators, and patients by increasing efficiency and decreasing costs.

## Author Contributions

LZ and HB shared the idea and supervised the study. WL collected policy documents from regulatory bodies and added the figures and tables. All authors were involved in drafting and revising the manuscript, reviewed, and approved the final manuscript.

## Conflict of Interest

The authors declare that the research was conducted in the absence of any commercial or financial relationships that could be construed as a potential conflict of interest.

## Abbreviations

NHP: Natural Health Product
NNHPD: Natural and Non-prescription Health Products Directorate
NPN: (Canada) Natural Product Number
SFDA: (Mainland China and Hong Kong) State Food and Drug Administration
ZZKZ: Zhizhu Kuanzhong.
