# Supplementary material for: A Comparative Study for License Application Regulations on Proprietary Chinese Medicines in Hong Kong and Canada
Source: Front Med (Lausanne). 2021 Mar 9;8:617625. doi: 10.3389/fmed.2021.617625 (PMC7985161; doi:10.3389/fmed.2021.617625)
Supplement: Supplementary file 1 [file Table_1.DOCX]

**Supplementary Material 1**

**The Quality** **Requirements of Proprietary Chinese Medicines in Hong Kong and Canada** **(13, 16)**

| **Requirements** | |
| --- | --- |
| **Hong Kong** | **Canada** |
| **A. Individual Product Quality Document** | |
| **1. Manufacturing Method**  Descriptions of the method  -according to each preparation step,  -processing procedure for each raw herb,  -names and quantities of all excipients used in the processes.  -for those procedures that may affect the quality of the Proprietary Chinese Medicine, the technical controls shall be specified e.g. number of hours and times required to boil the Chinese herbs | **1.** **Characterization**  (features and special qualities of a medicinal ingredient)  **A. Chemicals**  Highly purified ingredients described by the chemical name of the entire ingredient  **B. Processed ingredients**  (1) Process characterization of crude materials  (a) Crude materials: how they were obtained, harvested and cleaned  (b) Additional characterization: the processing after harvesting /purifying (i.e. dried or kept fresh, kept whole, cut or powdered)  (c) Characterization of identity, purity and stability of the crude material (especially when it is added to finished products directly without further processing)  (d) Characterization of live microorganisms includes culture conditions such as strain viability, specific media used for growth, growth temperatures, growth times, cell collection, etc.  (2) Process characterization for highly processed ingredients  (a) Characterization: full description of the process.  (b) Substances that are processed or chemically modified after purification of the active ingredient(s) are considered different medicinal ingredients from the original extract.  **C. Extracts**  (1) Standardized extracts  (a) Active constituent marker can be adjusted by standardization to a level of active constituent marker that is reproducible (naturally found in the plant or to a more concentrated level in an extract)  (b) Analytical marker (serves solely for analytical purposes and does not contribute to therapeutic activity) is adjusted to achieve a reproducible composition  (2) Fortified extracts: acceptable when the amount of component added is declared as a separate medicinal ingredient. |
| **2. Physicochemical Properties of Crude Drugs**  **(A)** **The crude drug(s) of a Proprietary Chinese Medicine that fall into any of the following four groups**, relevant literatures or scientific research reports detailing the physicochemical properties of the crude drug shall be submitted:  (1) A newly-discovered Chinese herb  (2) A new medicinal part of a Chinese herb  (3) An active group extracted from Chinese herb  (4) A set of active groups extracted from a compound prescription  **(i) Description**  Shape, size, color, texture, section surface, smell, etc. of all raw/crude herbs  **(ii) Identification Method**  (1) Microscopic Identification: observation of internal structure of the crude plant material through optical microscope or electron microscope  (2) General Physicochemical Identification: physicochemical analytical methods  (3) Chromatography: thin-layer chromatography, gas chromatography and high-performance liquid chromatography  (4) Spectrometry: ultraviolet and visible absorption spectrometry, infra-red spectrometry, etc.  **(iii) Inspection**  (1) Determination of foreign matters (organic and inorganic mix)  (2) Determination of ash (or acid insoluble ash)  (3) Determination of water  **(iv) Assay**  - conducted using chromatography, spectrometry or general physicochemical methods;  -analytical methods developed by the laboratory with details of the methodology involved, method validation etc.  - The manufacturer shall establish the permitted content level of active ingredient and marker compound in assayed items with supporting document.  - provide relevant information on testing conditions, selection criteria and the test report.  **(B) Crude herbs of the Proprietary Chinese Medicine that do not fall into any of the four groups** as mentioned above in (A), requirements are:  - literatures and articles on the physicochemical properties of the crude herb,  - specify the sources of the relating documents (such as copy of the monograph recorded in the Pharmacopoeia of the People’s Republic of China containing description, identification, inspection, assay of the herb), without having to submit the relevant test reports | **2. Physicochemical Properties of** **Raw Material**  **(A) Identification Tests of the ingredients**  Approach to and amount required is dependent on the type of ingredient and specific enough to distinguish the correct ingredient/plant species and plant part(s) from likely adulterants. Specific testing techniques for the substance and based on unique aspects of the ingredient at the raw material or finished product stage.  **(a) Appropriate Identification of botanical products**  (i) Macroscopic/organoleptic techniques: defined morphological and anatomical characteristics of the whole plant or plant part; colour, fracture, smell, taste, etc. (at raw material stage)  (ii) Microscopic techniques: to examine for characteristics established for the ingredient. (compared to authenticated or in-house reference materials and/or authoritative technical descriptions)  (iii) Chemical identification: includes chromatography, spectrometry, gravimetry, capillary electrophoresis, DNA fingerprinting, Fourier Transform Infrared spectroscopy, or Near-Infrared spectroscopy. Genomic, proteomic, and metabolomic studies, combined with statistical techniques such as Principal Components Analysis.  Use combination of botanical characteristics and chemical identification tests to eliminate misidentification of the botanical.  **(b) Appropriate identification of specific medicinal ingredients:**  Identify the Isolates and synthetic duplicates of materials of natural origin (at raw material stage): physical description and appropriate chemical identification tests.  **(c) Identity testing on the finished product**  Test for a specific medicinal ingredient if it is a single chemical entity; ease of testing is determined by the complexity of the matrix; description of the final dosage form documented as part of the identification; tests for identification include organoleptic evaluation (sensory characteristics e.g., taste, odour, feel, appearance such as colour and shape of the capsule or tablet, etc.); chemical identification tests where medicinal ingredient is defined chemical entity, or a marker is present; and physical description  **(B) Quantity**  **(a) Quantification by assay**  Botanical ingredients including extracts: specific marker compounds in whole herbs and extracts of botanical ingredients (by pharmacopoeial standard or appropriate limits set based on the data on safety and efficacy of the product and natural variability of the marker); quantitative tests for a particular component in an extract (at finished product or extract ingredient stage); if the evidence supporting a claim is based on the quantity of a particular active component, then quantification of that component should be performed at the finished product stage; the quantification to be recorded as 'potency' and the label tolerance limits should be set so there is an upper and a lower limit.  **(b) Quantification by input**  Means the active ingredients are not assayed at the finished product stage. The objective evidence that the quantity of a medicinal ingredient: has been added to the finished product is calculated using the manufacturing batch record controlled by appropriate application of GMPs and in-process controls; is expressed as the targeted weight of the processed substance in each unit of the dosage form; is appropriate for the ingredient; has comprehensive raw material specifications for the medicinal ingredient(s) to ensure adequate control of the medicinal ingredient(s) occurs; has standard operating procedures and batch records clearly document the controls during manufacturing to ensure adequate amount of medicinal ingredient is added to the mix during processing to achieve the labelled quantity per dosage unit. Target quantity for the medicinal ingredient (i.e., 100% of the label claim) should be indicated and controls on weight variation during tabletting or encapsulation included (5% variation in weight for individual dosages is acceptable)  **(C) Purity Standards**  Due to tests’ similarity, the purity standards requirements are listed in detail verses the safety requirements of Hong Kong in Appendix 2  **(D) Additional Tests**  **(a) General Indicators for Quality**  (i) Foreign matter: to ensure that the plant, algal or fungal material is entirely free from visible signs of contamination such as sand, glass and metal  (ii) Determination of acid insoluble ash: to determine the amount of inorganic impurities in the form of extraneous (non-biological) materials that are present in the materials.  (iv) Non-medicinal ingredients: all non-medicinal ingredie(iii) Water content: required for hygroscopic material; tolerance limits justified by data on the effects of moisture absorption on the product (e.g., potency and stability); a ‘loss on drying’ procedure, but some cases (e.g., plants containing essential oils) may need specific tests such as the Karl Fischer method  nts should adhere to any restrictions outlined in the NHPID and are used in quantities sufficient to support the intended purpose.  **(b) Performance Tests**  (i) Disintegration Times: specifications are required for solid oral NHPs intended to be swallowed whole, such as uncoated tablets, plain coated tablets or hard or soft gelatin capsules; official test method: DO-25 or other pharmacopoeial methods; tolerance limits based on the routine disintegration times for the product, but not more than 45 minutes for uncoated tablets or 60 minutes for plain-coated tablets; for rapidly dissolving NHPs (dissolution > 80% in 15 minutes at pH 1.2, 4.0, 6.8) that are highly soluble throughout the physiological range (dose/solubility volume < 250 ml from pH 1.2 to 6.8), disintegration testing may be substituted by dissolution testing (both tests are not required when it is to be chewed)  (ii) Dissolution**:** to measure the release of an active substance from solid oral dosage products (tablet or capsule); single-point measurements for immediate-release dosage forms; for modified-release dosage forms, follow appropriate sampling procedures under suitable test conditions; for extended-release NHPs, in vitro or in vivo correlation may be used to establish limits when human bioavailability data are available for formulations exhibiting different release rates  (iii) Uniformity of dosage units**:** refers to both the mass of the dosage form and the content of the active substance in the dosage form; specifications include one or the other, or both where the active constituent is less than 5% of the total weight; tolerance limits set for weight variation, fill volume or uniformity of fill.  **(c)** **Analytical testing and requirements to support label claims**  **-** Finished Product Specifications should include routine tests to support label claims such as “gluten free” or “sulphite free”  - a limit of 20 ppm for gluten and 20 ppm for sulphites for finished NHPs labelled as “gluten-free” and “sulphite free”.  **(d)** **Reduced testing schedules that are captured on specifications**  - The finished product specifications should clearly indicate the testing schedule with SOPs available for procedures in place.  - The implementation of the reduced testing program may be reviewed and verified during the life cycle of the site licencing process (should not compromise the safety of the product and is supported by a scientific rationale).  **(e)** **Antimicrobial effectiveness testing**  **-** Antimicrobial preservatives are ingredients added to dosage forms to protect them from microbiological growth and associated degradation, and tests must be done on the product to demonstrate the effectiveness of antimicrobial protection.  - Test methods and tolerance limits should be as specified in an acceptable Pharmacopoeia (e.g., current USP <51>; Ph. Eur. 5.1.3) and be performed on the final dosage form with suitable limits included.  - The preservatives’ concentration shown to be effective in the final dosage form should be below a level that may be toxic to human beings and at the lowest concentration necessary to preserve the product.    **Where an NNHPD monograph exists for an ingredient**, **the specification section of the monograph should be consulted** to determine whether or not specific outcomes for the ingredient have been indicated. The Natural Health Products Ingredients Database (NHPID) which lists additional specifications for various ingredients should be considered. |
| **3. Finished Product Specification, Methods and Certificate of Analysis**  **Product specification**   1. Description: color, shape, smell, etc. of the finished products 2. Identification: microscopic identification, general physicochemical identification, spectrometry and chromatography etc. which are specific, sensitive and reproducible 3. Assay: known chemical composition of other constituent or characteristic constituent in the prescription that can reflect the product quality; assay for any single chemical constituent or content level of all constituents or that of extracts; by referring to relevant literatures or be developed by the Product Licence holder/ manufacture’s own research stating methodology and provide references 4. Inspection: list all foreign matters in the finished products, clearly specified content levels of foreign heavy metals and toxic elements, pesticide residues, microorganisms, etc.; corresponding general tests for each dose forms with the clear product specifications   **Test Methods**  establish relevant test methods for each test specified in product specification; provide relevant basis including test conditions and selection criteria; accurately identify the finished products and reflect its quality.  **Test Report**  conducted according to the test methods; results in conformity with requirements specified in product specification. | **3. Finished Product Specification (FPS) and Test Methods**  **Product specification**  (1) Physical description  - Color, Shape, other tolerance limits as appropriate  (2) Identity testing on the finished product  - document the description of the final dosage form  - Tests might include tests: organoleptic evaluation (sensory characteristics e.g., taste, odour, feel, appearance such as colour and shape of the capsule or tablet, etc.); chemical identification tests e.g., comparison of a retention time of a High-Performance Liquid Chromatography (HPLC) peak with a standard (if the medicinal ingredient is a defined chemical entity, or a marker is present)  (3) FPS should include: ingredient specific test parameters; quantity; performance tests for specific dosage forms; tests and methods and tolerance limits for the microbial and chemical contaminants as outlined in the purity standard on Microbial Contaminants and Chemical Contaminants  **Test Methods**  - meet those set out in the List of NNHPD Recognized Methods  - must be Pharmacopoeial or other internationally recognized test methods. The following are currently considered acceptable in their entirety by the NNHPD: United States Pharmacopeia (USP); British Pharmacopoeia (BP); European Pharmacopoeia (Ph. Eur.); Pharmacopee francaise (Ph.f.); Pharmacopoeia Internationalis (Ph.I.); Japanese Pharmacopoeia (JP); or Food Chemicals Codex (FCC) |
| **4. Stability Test Report**  - Accelerated Stability Test for Group I application, to establish the stability and shelf life: regular assessment on changes in product quality when it is kept with temperature at 37℃-40℃ and relative humidity at 75±5%; the shelf life shall not be more than 2 years  - General Stability Test for Group I application, to re-assure the quality of the product remains stable: the product in its actual sales packaging is being kept under room temperature or proposed storage conditions of the product at the manufacturer. Regular inspections and tests are conducted to determine the expiration period of the drug.  - Real-Time Stability Test (for Group II and Group III application, to ensure the safety and efficacy of clinical application of the product within its shelf life: regular assessment on changes in product quality to determine the stability and shelf life of the product when it is in its actual sales packaging kept in ambient conditions (i.e. with temperature at 25℃±2℃ and relative humidity at 60%±5%). | **4. Stability Test**  Stability Testing:  - to establish a shelf-life for the NHP  - to assess the impact of environmental factors (temperature, humidity, light, etc.),  - to assess the packaging material (the container closure system), and  - to assess intrinsic factors (ingredient interactions, degradation, natural spoilage etc.) on the quality, safety and efficacy of the product.  The product licence holder is ultimately responsible for product quality, and as such, product stability.  If these obligations are delegated to a third-party a clear understanding of roles and responsibilities must be established to ensure that compliance is achieved.  The NNHPD may request stability information as a component of the site licensing process (Refer to the Good Manufacturing Practices guidance document on stability). |
| **B. Requirements for test laboratories** | |
| **1. Met the requirements set by the International Standardization Organization**  Accepted Institution which issued requirements to regulate laboratories conducting tests: ISO/IEC 17025; Good Laboratory Practice; any other laboratories that are accepted by the Chinese Medicines Board; or other municipal Institutes for Drug Control in China that are recognized both by the State Food and Drug Administration and the Chinese Medicines Board | Referenced to:  - Good Laboratory Practice |
| **2. Others accepted** **Certificates of analysis and stability test reports:**   - Issued by laboratories with ISO/IEC 17025 accreditation of the 3 basis tests: Heavy metals and toxic element test; Pesticide residues test; and Microbial limit test - For stability tests, at least the first batch of product should be conducted in test laboratories that have met the above requirements. The remaining batch(es) can be conducted by the manufacturer of the product, which met the Good Manufacturing Practice, for both manufacturing process and quality control of Proprietary Chinese Medicines |  |
