# Supplementary material for: A Comparative Study for License Application Regulations on Proprietary Chinese Medicines in Hong Kong and Canada
Source: Front Med (Lausanne). 2021 Mar 9;8:617625. doi: 10.3389/fmed.2021.617625 (PMC7985161; doi:10.3389/fmed.2021.617625)
Supplement: Supplementary file 2 [file Table_2.DOCX]

**Supplementary Material 2**

**The Safety Requirements and Purity Standards (under Quality Requirements’ guidelines) of Proprietary Chinese Medicine in Hong Kong and Canada respectively (14, 16)**

| **Hong Kong (Safety Requirements)** | **Canada (Purity Standards)** |
| --- | --- |
| Documents supporting the safety claims of the product are required to be submitted to the Chinese Medicines Board for assessments. The documents shall include the basic and toxicological tests. Other reference material may be provided to support the safety claims of the products, e.g. the published bibliography or monographs, etc.  Below are the tests required: | Safety Requirements for Proprietary Chinese Medicine under:  - Traditional Medicine is supported by its history of use (with evidence of at least 50 consecutive years of traditional use within a cultural health system or paradigm) and no new, unknown safety concerns have been identified by the findings outside of evidence for traditional use. Two independent references supporting safety based on belief/theories, experiences within a single system of traditional medicine. When scientific evidence suggests a risk, scientific evidence should be provided to substantiate safety.  - Modern Health Claims are based on the identified risks to health, such as the severity and seriousness of adverse effects. Evidence recommendations are categorized into low, medium, and high risk. Within any risk category, the evidence may be sufficient to support both safety and efficacy when it is appropriate for the claim and when it fully reflects the product’s recommended conditions of use. For the low and medium categories, methodologically weak safety evidence should be supplemented to demonstrate consistency in results and plausibility. For high risk category, product specific evidence is recommended with a complete critical summary reflecting the totality of evidence that usually reflect more than one type of evidence.  Due to the test requirements’ similarity, in this comparative study we compare the safety requirements of Proprietary Chinese Medicine product in Hong Kong to the product’s purity standard in Canada. |
| **A. Heavy metals and toxic element test**  Heavy metals (mercury, lead and cadmium) or toxic elements (arsenic) contained in Proprietary Chinese Medicines: may be a result of environmental pollution of the raw herbs, from contamination in the preparatory process; or simply due to the Chinese herb(s) originally contained heavy metals or toxic elements as ingredients is/are formulated in the preparation, as such, the following requirements should be referred:  **(1) Established medicines category**  The quantity of the Chinese herb used must strictly adhere to the amount specified in the Pharmacopoeia of the People’s Republic of China, etc., and the product meets the processing requirements for processing and production, in the course of manufacturing.  **(2) Health-preserving medicines in the non-established medicines category**  As products may be used long term, Chinese herb originally contained heavy metal(s) or toxic element(s) should not, generally be used in these products.  **(3) Single Chinese medicine granules in the non-established medicines category**  It is recommended that no Chinese herb originally contained heavy metal(s) or toxic element(s) should be used in these products.  **(4) New Medicines Category**  Comprehensive test reports under the New Medicines Category are required to support the product’s efficacy and safety.  Permitted level for heavy metals and toxic element in Proprietary Chinese Medicines:  Element Max. Total Intake  Arsenic 1500 micrograms/day  Cadmium 3,500 micrograms/dosage  Lead 179 micrograms/day  Total mercury 36 micrograms/day | **A. Chemical Contaminants**  **(1) Elemental impurities**  Catalysts and environmental contaminants that may be present in raw materials or finished products, which:  - may occur naturally,  - be added intentionally as part of the manufacturing process, or  - be introduced inadvertently (e.g., through interactions with processing equipment).  - may be tested individually, or as total heavy metals expressed as lead at the finished product stage or at the raw material stage if all medicinal and non-medicinal ingredients are tested.  Finished product testing is not required if testing was conducted on the raw materials or if the ingredients meet an appropriate pharmacopoeial grade.  **(2) Topical products**  Heavy metal concentrations in topical products should meet the following limits:  Element Limit in parts per million (ppm)  Arsenic 3 ppm  Cadmium 3 ppm  Lead 10 ppm  Total mercury 1 ppm  Antimony 5 ppm |
| **B. Pesticide residues test**  **Requirements (1)**  May choose either the finished product or individual crude drug to test for the 9 types of organochlorine pesticides. If necessary (e.g. when the manufacturing methods of the Proprietary Chinese Medicine cause condensation of the remaining pesticides in the finished products), the Chinese Medicines Board may require the test to be conducted on the finished product.  **Requirements (2)**  Reports on the test methods and results are required, and the test results must not exceed the maximum permitted level as specified by the Chinese Medicines Board. | **B. Pesticide residues**  - Test done according to the multi-residue method and limits outlined in the Ph.Eur., United States Food and Drug Administration’s Pesticide Analytical Manual 1 or WHO Methods for Pesticide Screening l.  - Pesticides used in treatment of the plant or where residues are suspected and may carry over to the final dosage form should be tested.  - Pesticide testing may not be required for some products, such as products with a certified organic content of ≥95% or topical products using pharmacopeial grade ingredients that do not have pesticide residue limits.  - Testing of chemical residues in accordance with the Food and Drug Regulations is acceptable if the ingredients are also used as foods.  - Pesticide limits for specific food commodities are found in the List of Maximum Residue Limits Regulated Under the Pest Control Products Act. |
| **C. Microbial limit test**  The product shall be assessed in respect to its dose form and will be considered pass only when it meets all the three criteria set**:**  (1) Total aerobic count  (2) Moulds and yeast count  (3) The presence of specified bacteria | **C.** **Microbial Contaminants**  Requirements for specific products and routes of administration  **Multi-Component products**  - For products with multiple ingredients, each with different tolerance limits, the tolerance limit for the finished product would be based on the least stringent limit;  - limits should be reduced if warranted by routine analysis showing lower levels of contamination.  - For products containing live microorganisms, a method of enumerating viable members of the family, Enterobacteriaceae should be used, or the Health Canada test MFLP-43 "Determination of Enterobacteriaceae". Selective testing for coliforms or only for members of the genus Enterobacter is not sufficient as it may potentially fail to screen for other gram negative facultative rods that belong to the same family of Enterobacteriaceae and are also known pathogens such as members of the genera Klebsiella, Shigella, etc.  **Products in liquid dosage form**  Pseudomonas aeruginosa testing is generally required for non-probiotic liquid products unless alcohol is present at a concentration greater than 50%. |
| **D. Acute toxicity test**  Presented in its median lethal dose (LD50). Presented in its maximum tolerable dose (MTD) if LD50 is not obtainable.  **(1) Median lethal dose (LD50):** The dosage which can cause death of half of the test animals in single administration.  **(2) Maximum tolerable dose (MTD):**  The highest dose that the test animals can tolerate without causing death. The dose may be administered to the animals once or multiple times in a day. | **D. Other Impurities**  **(1) Mycotoxins (e.g., aflatoxins) Testing**  It is required for products containing ginseng or peanuts or any substance derived from these sources as they may be contaminated with aflatoxins due to poor agricultural practices and storage conditions, and if a medicinal ingredient has documented cases of fungal contamination or if fungal contamination is considered likely.  The NNHPD has set tolerance limits of < 20 μg/kg (ppb) for aflatoxins (B1+B2+G1+G2), and <5 μg/kg (ppb) for aflatoxin B1.  **(2) Cyanobacterial toxins (e.g. microcystins)**  NHPs containing the cyanobacterium (blue-green alga, BGA), Aphanizomenon flos-aquae, have a history of contamination with a group of hepatotoxic compounds called microcystins (MCs) so testing for microcystins is necessary.  The NNHPD has adopted a finished product tolerance limit of 0.02 μg MC-LR/kg b.w./day or a raw material tolerance limit of 1 ppm, provided that the total consumption per day remains less than 0.02 μg MC-LR/kg b.w. when calculated for the finished product.  **(3) Solvent residues**  - Solvents known to cause unacceptable toxicities (ICH Class I) are not considered appropriate for NHPs and if it cannot be avoided, then confirmation of acceptability of the solvent is required before it is used on the raw material.  - Use of solvents with less severe toxicity (ICH Class II) should be limited in order to protect consumers from potential adverse effects.  - Wherever possible, the least toxic solvents (ICH Class III) should be used.  - Testing for solvents should be done according to Pharmacopoeial (USP, Ph.Eur.) methods using gas chromatography (GC) and high-performance liquid chromatography (HPLC) techniques. Tolerance limits for solvent residues should conform to ICH or pharmacopoeial limits.  - If only ICH Class III solvents are used in the manufacture of the NHP, a test for loss on drying with a tolerance limit of not more than 0.5% is considered acceptable to test for solvent residues.  If manufacturers have ascertained through a cumulative procedure, according to USP <467>, that the level of residual solvents is acceptable and below the acceptable limit, residual solvent resting is not required.  **(4) Hormone testing of animal materials**  - Non-human animal materials used in NHPs, such as ovaries, hypothalamus etc., must not contain sex hormones (progesterone, estrogens, testosterone, and DHEA), which are regulated as prescription drugs under the Food and Drug Regulations or as controlled substances as set out in Schedule IV to the Controlled Drugs and Substances Act, and thus are excluded from NHPs by the Natural Health Products Regulations.  - Hormone testing is only required for those animal materials used in NHPs that are known to contain hormones regulated in Canada as prescription drugs or as controlled substances.  **(5) Incidental impurities, related substances and process impurities**  Processing or purification steps may introduce organic or inorganic impurities in the product. All known impurities present in the raw material at significant levels should be listed on the raw material specifications with their associated tests and tolerance limits. If the impurity profile of an isolated or synthetic medicinal ingredient is altered due to a change in the source material or manufacturing process, revised specifications with the new tolerance limits for the impurities should be submitted to the NNHPD.  **(11) Potential adulterants in natural health products**  The finished product should be free from adulteration. The potential risk that undeclared ingredients are present should be considered along the entire supply chain.  **(12) Ingredients sourced from tissues that are susceptible to transmissible spongiform encephalopathy (TSE) and bovine spongiform encephalopathy (BSE).**  - All products should be free from TSE and BSE causing agents.  - Not to use tissues that are susceptible to TSE including bones (other than vertebral column or skull) of cattle, sheep, goat, deer or elk velvet antlers.  - Alternatives such as plant materials (e.g. vegicaps), gelatin made of materials from animals that are not susceptible to TSEs (e.g. pig), or gelatin made from skin and hides of any animal are suggested. |
| **E. Long-term toxicity test**  To provide reference for determining a safe dose level  Based on the clinical administration period, set the appropriate test period and administer the product to test animals continuously during this test period. Observe and record the animals’ toxicity reactions, including their (first) reactions and seriousness when intoxication occurs, also the development and recovery of their tissue damage and organ dysfunction after the administration is stopped. |  |
| **F. Local toxicity test**  To examine whether local-applied medicines will cause any irritation or allergic reaction  **(1) Local dermal toxicity Test** (a) Skin Irritation Test: after administering the test medicine to both intact skin and wounded skin, conduct regular observation with the naked eye and histopathologic examinations to evaluate the intensity of the skin irritation.  (b) Skin sensitivity test: to find out whether the medicine, after being repeatedly applied on animal’s skin, will cause the animal’s immune system to over-react so that allergic reaction would appear when the skin comes into contact with the medicine again.  **(2) Mucous membrane irritation test**  After applying the medicine to certain mucous membrane (according to clinical administration route), observe the irritation reaction of test animals and the recovery process regularly. |  |
| **G. Mutagenicity test**  For Group II and Group III application only, to examine whether the Proprietary Chinese Medicine has carcinogenicity or reproductive toxicity  **(1) Bacterial reverse mutation test**  Observe and record the reverse mutation induced by the test medicine in specified microbial species, so as to judge whether it has positive relation with the test medicine.  **(2) Chromosomal aberration test with mammalian cells in culture** Observe whether the test medicine has induced any aberrance in mammalian cells in culture and record the occurrence rate so as to judge whether it has positive relation with the test medicine.  **(3) Micronucleus test with rodents** Count the number of cells with micronucleus of the treated animals, so as to judge whether it has positive relation with the medicine. |  |
| **H. Carcinogenicity test**  For Group II and Group III application only, to examine whether the test medicine or its metabolites has carcinogenicity or tumorigenicity  **(1) Preliminary carcinogenicity study** To determine the highest dose level that can be used in the full-scale carcinogenicity study.  **(2) Full-scale carcinogenicity study** Observe and record the occurrence rate of tumor in test animals after long-term administration and assess whether the test medicine has carcinogenic activity on the animals. |  |
| **I. Reproductive and development toxicity test**  For Group II and Group III application only, to examine whether the test medicine has toxic effects on animal’s reproductivity and has teratogenic effect on their offspring  **(1) General reproductive toxicity test** Conducted prior to and in the early stages of pregnancy to examine whether the product is toxic to the reproductive system of the test animals.  **(2) Teratogenicity test** To examine the toxicity of the medicine on the organogenesis of the fetus.  **(3) Perinatal toxicity test**  To examine the toxicity of the medicine and is conducted during perinatal and lactation periods. |  |
| **J. Requirements for test laboratories**  Laboratories conducting product safety tests for Proprietary Chinese Medicines should have met the requirements set by the International Standardization Organizations, i.e., ISO 17025,"Good Laboratory Practice" (GLP) or any other laboratories that are accepted by the Chinese Medicines Board. Other municipal Institutes for Drug Control in China that are recognized both by the State Food and Drug Administration (SFDA) and the Chinese Medicines Board will also be accepted. | **E. Requirements for test laboratories**  Referenced to:  - Good Laboratory Practice (GLP) |
| **K. Summary report of product safety documents**  Summary report of product safety documents - to give an overall conclusion and a reasonable assessment on the safety of the Proprietary Chinese Medicine.  The applicant should draw the conclusion based on the product safety documents submitted. | **F. Summary report of product safety documents**  A Safety Summary Report is not required for Compendial application, and for all other types of product licence applications, the Safety Summary Report in accordance with the recommended conditions of use should consist of the following 2 sections:  - Safety Overview to provide a summary of all relevant safety information related to the NHP, including the following: known adverse reactions associated with its use (including adverse reaction reports, if available); preclinical or clinical toxicology; previous marketing experience; interactions (e.g. with other medicinal products, foods, standardized laboratory tests); in-text references  - Risk Information and Risk Mitigation to provide the following: risk information, including cautions, warnings, and contraindications associated with the use of the NHP for self-care; strategies to mitigate any risk(s) associated with the use of the NHP for self-care; and in-text references |
