# Supplementary material for: A Comparative Study for License Application Regulations on Proprietary Chinese Medicines in Hong Kong and Canada
Source: Front Med (Lausanne). 2021 Mar 9;8:617625. doi: 10.3389/fmed.2021.617625 (PMC7985161; doi:10.3389/fmed.2021.617625)
Supplement: Supplementary file 3 [file Table_3.DOCX]

**Supplementary Material 3**

**The Efficacy Requirements of Proprietary Chinese Medicines in Hong Kong and Canada (15, 16)**

| Requirements | | |
| --- | --- | --- |
| **Hong Kong** | **Canada** |  |
| **1.** **Reference materials on product efficacy**  Including reference literature or documentary proofs on long history of use.  The requirements are listed as follows:  (i) Established medicines category  - For Proprietary Chinese Medicine that is formulated in according to an ancient prescription, a modified ancient prescription or pharmacopoeia prescription, or any other prescription from National Drug Standards of the People’s Republic of China, the applicant shall submit copies of relevant materials from Chinese medicines bibliography, Pharmacopoeia or any other National Drug Standards of the People’s Republic of China.  (ii) Non-established medicines category  - Health-preserving medicines: the claimed therapeutic functions shall be supported by research studies, or the functions of which have been described in health care literatures compiled by Chinese medicines professionals.  - Single Chinese medicine granules: copies of relevant materials from Chinese medicines bibliography or Pharmacopoeia shall be submitted.  (iii) New medicines category  - Reports on product efficacy evaluation and clinical trials, etc. are necessary. | **1. Evidence requirements for safety and efficacy**  The safety and efficacy of health claims associated with NHPs must be supported by appropriate evidence such as clinical trial data to references to published studies, journals, pharmacopoeias and traditional resources. The type and amount of supporting evidence required is dependant on the proposed health claim of the product and its overall risks.  It is the responsibility of the applicant to provide evidence demonstrating that safety (risk) has been established and any risks sufficiently mitigated; that efficacy (benefit) has been demonstrated; and that quality is supported.  The evidence requirements for efficacy are listed depending on whether the product is a:  - Traditional Medicine;  - NHP with Traditional use claims or with Modern Health Claims |  |
| **2. Interpretation and principle of formulating a prescription**  The efficacy and safety of a Proprietary Chinese Medicine is dependant on whether the selected prescription has clearly defined indications or functions, reasonable formulation, correct composition and appropriate dosages.  The contents should include the analysis based on the theory of Chinese medicine i.e. the roles of ‘the principal, assistant, adjuvant and guiding drugs’ in the prescription, brief description on the properties, flavors, channel tropism, functions and indications of each drugs, and hence to interpret the efficacy of the Proprietary Chinese Medicine.  The interpretation and principle of formulating a prescription shall be written by professionals.  General requirements on interpretation and principle of formulating a prescription:  Source:  The provenance of the prescription should be clearly stated with copies of relevant bibliography for verification. Further elaboration and interpretation may be required if the prescription is an empirical prescription, a modified ancient prescription or a newly developed prescription.  Ingredients:  Includes name, quantity and processing method of all ingredients of the prescription.  Usage and Dosage of the preparation:  Shall be specified.  Functions and indications:  The therapeutic functions and indications of a prescription should be: interrelated; descriptions should highlight its therapeutic effects and be accurate and comprehensive; and the description of indications should include clearly defined syndromes and refer mainly to syndromes in Chinese medicine theory and be expressed in academic terminology of Chinese medicine.  Interpretation:  - An analysis and description on functions, indications of the prescription, interaction and compatibility of each ingredient should be given.  - Interpretation of a prescription can start with the Chinese medicine syndromes – elaboration on etiology, pathogenesis, characteristics of the syndrome, and syndrome differentiation and establishment of therapeutic principles. Then, interaction and compatibility analysis on the basis of the “principal, assistant, adjuvant and guiding ingredients” in the prescription.  - In the description, the properties, flavours, functions, status and actions of each ingredient in the prescription should be well explained.  Precautions (if any):  Should contain cautions when using the prescription, for instance: contraindications or patients for whom this prescription is not suitable. | **2. Efficacy Evidence for Traditional Medicines**  Traditional use claims are divided into two sub-categories according to the evidence provided:  - pharmacopoeial evidence alone; or  - other types and/or combinations of references supporting traditional use.  Applicants to provide a cover letter with their applications that clearly indicates which of the two evidence categories their product is intended for.  **A. Pharmacopoeial Evidence for Traditional Medicines**  (a) Products providing only pharmacopoeial evidence and answering “yes” to all elements of the Checklist for the Traditional Pharmacopoeial Evidence Category only require one supporting reference (Answering “no” to any of the questions posed on this checklist excludes the product from being assessed within the pharmacopoeial stream).  (b) provide one of the following as evidence in supporting the claim:  - A copy of the relevant pages of a monograph from a recognized pharmacopoeia (e.g., The Pharmacopoeia of the People’s Republic of China); or  - A copy of a monograph published by a reputable agency with a definition of traditional medicines comparable to that of the NNHPD (e.g., translated version of the Drug Standard of People’ Republic of China);  (with an English or French translation of the relevant monograph pages)  (c) The information on Part 4 of the Product Licence Application form (e.g., medicinal ingredients, route of administration, dose, duration of use, method of preparation, etc.) should be comparable to that stipulated in the supporting monograph or reference. As an exception, when a monograph specifies an indication related to a serious disease, condition, or abnormal physical state (e.g., cancer, depression, alcoholism), revisions are permitted to make the claim more appropriate for a non-prescription product.  (A multiple ingredient rationale and the Checklist for the Traditional Pharmacopoeial Assessment Evidence Category are not required when pharmacopoeial evidence is provided)  **B. Other Types of Efficacy Evidence for Traditional Medicines**  - Products providing more than pharmacopoeial evidence as the evidence supporting efficacy or answering “no” to any of the elements of the Checklist for the Traditional Pharmacopoeial Evidence Category should provide at least two independent references that support the recommended conditions of use.  - Independent references are those that do not cite the same source, or each other, as the main source of information regarding the traditional use of the ingredient.  - The references should be authoritative and from a reputable source as determined by NNHPD.  - In the case where only one written reference exists or where multiple references refer back to a single original source, an expert opinion based on practitioner experience and knowledge may be considered as a possible substitute for a second reference. |  |
| **3. Principal pharmacodynamic studies**  For Group III application only, to obtain preliminary verification on the therapeutic effects of the Proprietary Chinese Medicine, and to determine the potency, extent of effects and properties of the Proprietary Chinese Medicine, thus providing fundamental but important information for further clinical investigations.  (i) Brief requirements  - With the theory of Chinese medicine, principal pharmacodynamics studies of Proprietary Chinese Medicines make use of modern scientific methods to draw up trial protocols with Chinese medicine characteristics.  - According to the claimed therapeutic functions and indications of the Proprietary Chinese Medicine, appropriate animal disease models and trial methodology are designed to evaluate its therapeutic effects and to provide a scientific basis for these claims.  (ii) Requirements for test laboratories  - Laboratories conducting tests on the studies must have met the requirements set by the International Standardization Organization (i.e. ISO/IEC 17025), Good Laboratory Practice (GLP), or any other laboratories which are accepted by the Chinese Medicines Board. Other municipal Institutes for Drug Control in China that are recognized both by the State Food and Drug Administration (SFDA) and the Chinese Medicines Board will also be accepted. | **3. Efficacy Evidence Recommendations for NHPs with Modern Health Claims**  Efficacy evidence should support:  - the reasonable association of the medicinal ingredient(s) with the health claim(s) and demonstrate that therapeutic efficacy of the product will be supported by at least one medicinal ingredient or the combination of more than one, with respect to the specific target population intended, the specific directions of use and the specific system of medicine, when appropriate.  - the health context of the product and provide enough background information to describe the characterization of the health condition implied by the claim and the health context of the recommended use.  - both safety and efficacy when it is appropriate for the claim and when it fully reflects the product’s recommended conditions of use.  The minimum type of evidence from a higher risk category may be used to support a claim in a lower category as long as it is appropriate for the condition. For the low and medium categories, methodologically weak efficacy evidence should be supplemented to demonstrate consistency in results and plausibility.  **A. Efficacy Evidence for the High-Risk Category**  NHPs making claims for the treatment, prevention or cure of serious health conditions should meet the evidence criteria for the high-risk category.  - The evidence package should include a complete critical summary reflecting the totality of evidence.  - Evidence should be presented in the form of a systematic review outlining the validity and causality elements for each reference by providing a critical analysis of the study design types, and the quality and quantity of each evidence type that supports and refutes the claim. Product-specific evidence is recommended.  - Evidence provided should demonstrate statistically significant outcomes, clinically meaningful differences, relevance to the target population, and overall consistency of the results across all studies of acceptable quality. Data should support the characterization of the disease.  - Additional evidence to support interactions and a complete summary reflecting the totality of evidence should be provided other than the below evidence:   - Phase III or phase IV clinical trials, randomized, controlled and well-designed (For treatment, cure, and prevention claims or for health support claims when they imply treatment, cure, prevention, and risk reduction claims if the study is not multi-centred, at least two studies are required). - Meta-analysis, controlled and well-designed (conclusions should be based primarily on phase III trials, not phase II trials; primary evidence may be requested). - Prospective observational studies or combinations of one prospective study and one retrospective study (evidence only meets minimum requirements for prevention and risk reduction claims) - Evidence of a positive decision from another regulatory agency (Documentation in the form of an authorization letter or positive decision must be submitted that includes details on what was approved. A description of the regulatory requirements from the other regulatory agency should be provided   **B. Efficacy Evidence for the Medium Risk Category**  NHPs making claims for major health conditions and diseases should meet the evidence criteria of the medium risk category. - The evidence for products or ingredients in this category can be submitted as individual references, although additional information or evidence is recommended to help support: the recommended conditions of use, the health context of the product, and the comparability of the ingredient forms.  - Evidence are:   - Systematic review other than meta-analysis (Conclusions should be based primarily on phase III trials, not phase II trials; primary evidence may be requested) - Published, peer-reviewed, detailed narrative reviews which cite detailed primary evidence (Detail should include: defining characteristics of the ingredient; primary endpoints/outcomes with statistical and clinical significance; the studied sub-population’s age, gender, and health state; the dosing regimen and dosage form; the route of administration; the directions of use; any restrictions to study entry of participants based on interactions/risk; any identified adverse reactions) - Phase II clinical trials (Two pieces of evidence of equivalent ranking or higher are required to support efficacy. When the evidence provided to support the claim is methodologically weak, it should be supplemented to demonstrate consistency in results and plausibility) - Epidemiological studies (Evidence only meets minimum requirements for prevention and risk reduction claims. Two pieces of evidence of equivalent ranking or higher are required to support efficacy) - Published compilations referring to traditional use (Evidence can be used to support safety only)   - Evidence should ideally demonstrate: a well described study population; a record of the flow of subjects through the trial; power analysis to determine proper number of subjects; random allocation; blinded assessment of outcome; intention to treat analysis; should usually be assessed compared to current standard therapy; and in addition to validity, evidence should demonstrate reasonable causality supporting the efficacy of the product.  **C. Efficacy Evidence Requirements for the Low Risk Category**  The low risk category include NHPs making claims for minor health conditions and diseases; NHPs for the treatment of symptoms or risk factors of serious or major conditions or the risk reduction of these conditions; and NHPs for general health maintenance, support, or promotion that refers to modification of a biochemical or physiological function of a nutritional nature or implies benefit to a minor disease or health condition.  Evidence requirements reflect the low risk nature of these products but should still demonstrate key aspects of validity and be appropriate for the recommended conditions of use.  - Evidence are:   - Phase II clinical trials (One piece of evidence of equivalent ranking or higher is required to support efficacy. When the evidence provided to support the claim is methodologically weak, it should be supplemented to demonstrate consistency in results and plausibility) - Epidemiological studies (Evidence only meets minimum requirements for prevention and risk reduction claims. One pieces of evidence of equivalent ranking or higher are required to support efficacy) - Pilot and open label studies (Two pieces of evidence of equivalent ranking are required to support efficacy. The two different studies may be of equivalent or higher ranking. When the evidence provided to support the claim is methodologically weak, it should be supplemented to demonstrate consistency in results and plausibility) - Reputable textbooks (Textbook should reflect human in vivo data if the ingredient is an essential nutrient) - Demonstration of food use (Evidence can be used to support safety only)   This category includes most vitamins, minerals, essential nutrients, and other nutrients recommended for use by healthy adults. These types of ingredients are often associated with NNHPD pre-cleared information. |  |
| **4. General pharmacological studies**  For Group III application only, to observe and identify other pharmacological effects in addition to the principal therapeutic actions of the Proprietary Chinese Medicine.  (i) Brief requirements: in the studies, drugs are administered to the test animals to observe its effects on the animals’ nervous system, cardiovascular system as well as respiratory system.  (ii)Requirements for test laboratories  - Laboratories conducting the studies must have met the requirements set by the International Standardization Organization (i.e. ISO/IEC 17025), Good Laboratory Practice (GLP), or any other laboratories which are accepted by the Chinese Medicines Board. Other municipal Institutes for Drug Control in China that are recognized both by the State Food and Drug Administration (SFDA) and the Chinese Medicines Board will also be accepted. |  |  |
| **5. Clinical trial protocol and summary report**  For Group III application only. Clinical trial refers to trial with humans as test participants. Under controlled conditions, the safety and efficacy of the product are examined and assessed in a scientific manner.  (i) Brief requirements  (a) Phases of clinical trials  Clinical trials studies are divided into Phase I，II，III and IV with details described below:  Phase I  A preliminary evaluation on clinical pharmacological effect and safety profile Phase I of the test medicine in humans. It is to observe the degree of tolerance in human to the new drug, whenever technically possible, pharmacokinetic studies shall be conducted as to get an indication of the dose schedules that might be used in subsequent studies.  Phase II  Aim to assess the efficacy and safety of the test medicine, thereby to determine the clinical administration dosage.  Phase III  It involves multi-center studies and is essential to further evaluate the efficacy and safety of the test medicine  Phase IV (Upon approval of the registration)  Post-marketing surveillance is to monitor the efficacy of the product and to detect any adverse reaction after it is available for general use.  (b) Contents of clinical trial protocol  - Clinical trial protocol should include: Title, foreword, trial objectives, establishment of trial syndromes, inclusion criteria for study subjects, number of trial subjects needed to achieve the trial objective (based on statistical considerations), trial methodology and proposal, treatments, observation items and their methods, assessment of therapeutic effects, observations, assessment and reports on adverse reaction or events, criteria for trial subject to terminate the trial, methods of data processing and analyzing, design of the case report form, investigation of compliance, participant’s informed consent form, qualification and responsibility of investigators, qualification and responsibility of monitors, management of trial drugs, etc.  (c) Contents of the summary report of clinical trials  - The summary report shall summaries and analysis the information collected in the trials.  - When the trials are finished, each trial center shall compile and submit their own trial reports, and the management in charge of the overall clinical trial shall then compile a summary report summarising the trial reports submitted by each center.  - The summary report shall include title, summary, objectives, case selection, trial methodology, assessment on therapeutic effects, trial results, typical cases, analysis and explanation on withdrawn cases or of adverse events, discussion and conclusions on therapeutic effects and safety. In addition, names, professions and titles of the protocol designer, investigators of the clinical trial and the principal investigator(s) shall also be included.  - In the discussion section of the summary report, the conclusions shall cover the functions (or indications), applicable scope, administration regimen, treatment period, therapeutic effect, safety, adverse reaction, contraindications and other caution information of the new drug based on the results of the trial.  (d) Documents to be submitted upon application  - Upon application, the applicant shall submit: the clinical trial protocol of all phases, approval letter from the ethics committee, and the summary report of phases I, II and III of the clinical trial. Within 2 years after the registration, the applicant shall submit the summary report on phase IV of the clinical trial.  (ii) Requirements for Clinical Trial centres  - Centres conducting the clinical trials for Proprietary Chinese Medicines must have met the requirements of “Good Clinical Practice for proprietary Chinese medicines” (GCP) or other equivalent standards. Other clinical trial centers in China that are recognized both by the State Food and Drug Administration (SFDA) and the Chinese Medicines Board will also be accepted. |  |  |
| **6. Summary report on product efficacy documents**  To give an overall conclusion and a reasonable assessment of product efficacy.  Brief requirements  - The applicant shall organize and compile a summary on the product efficacy documents where upon he/she shall present a reasonable and well-founded evaluation and conclusion on the efficacy of the Proprietary Chinese Medicine | **4. Summary report on product efficacy documents**  The Evidence Summary Report consists of the following 3 sections:   - Recommended Use or Purpose (health claim) to provide: the proposed health claim as indicated; the type of claim (treatment, risk-reduction, structure-function or non-specific); and references that support the claim; - Critical Overview should be organized based on the claims of the product to provide: a critical analysis and summary of the totality of evidence from all relevant sources of evidence pertaining to the use of the NHP according to the recommended conditions of use; pharmacokinetic and pharmacodynamic information where available (human data preferred, animal or in vitro where necessary), to support its safety and/or efficacy; and in-text references. - Dosage and Other Conditions of Use to provide information to support the recommended conditions of use indicated (with reference): dose (amount, frequency and directions of use, including reference to the Additive Combinations Evaluation Form if appropriate); dosage form; route of administration; and duration of use (if any). |  |
